# The 12‐Lead Electrocardiogram of the Rugby Football League Athlete: Impact of Sex and Age

**DOI:** 10.1002/ejsc.12304

**Published:** 2025-05-05

**Authors:** Callum Warrington, Andy Thompson, Jade Read, Jonathan Abram, Nathan Mill, Robert Cooper, Keith George, David Oxborough

**Affiliations:** ^1^ Research Institute for Sport and Exercise Sciences Liverpool John Moores University Liverpool UK; ^2^ St Helens Rugby Football League Club St Helens UK; ^3^ Liverpool Heart and Chest Hospital Liverpool UK

**Keywords:** age, athletes heart, electrocardiogram, sex

## Abstract

Electrocardiogram (ECG) findings in male Rugby Football League (RFL) athletes have previously been investigated but variations in other demographics are less understood. The study examined 161 ECGs in male and female, paediatric (< 18 years old) and adult (≥ 18 years old) RFL athletes. Athletes (65% male, 35% female) aged 14–33 years underwent a 12‐lead ECG that was assessed for training‐related cardiac electrical adaptations. Four athletes with abnormal ECGs were excluded. Results indicated that male athletes had an increased PR interval (152 ± 32 vs. 140 ± 19 ms; *p* < 0.001), QRS duration (98 ± 7 vs. 87 ± 6 ms; *p* < 0.001), voltage criteria for right (6 ± 3 vs. 4 ± 2 mm; *p* < 0.001) and left (33 ± 9 vs. 27 ± 7 mm; *p* < 0.001) ventricular hypertrophy (RVH/LVH) compared to females, who exhibited increased resting heart rate (HR) (67 ± 11 vs. 62 ± 11 bpm; *p* < 0.001) and QTc intervals (408 ± 45 vs. 398 ± 22 ms; *p* = 0.028). Adult athletes had a reduced HR (61 ± 10 vs. 66 ± 13 bpm; *p* < 0.001) and RVH criteria (5 ± 2 vs. 7 ± 3 mm; *p* = 0.015) compared to paediatric athletes. When controlling for weekly training hours in adult athletes, males present only a longer PR interval (156 ± 32 vs. 141 ± 19 ms; *p* < 0.037) and QRS duration (99 ± 7 vs. 87 ± 7 ms; *p* < 0.001) when compared to females. These results highlight the need for tailored cardiac screening guidelines that account for an RFL athlete's sex, age and training exposure.

**Summary**

- Significant differences in ECG parameters between male and female Rugby Football League (RFL) athletes are present, with males demonstrating a longer PR interval and QRS duration.
- Significant differences in ECG parameters are also observed between adult age group and paediatric age group RFL athletes. With paediatric athletes presenting with higher resting heart rates and increased criteria for right ventricular hypertrophy.
- 12‐lead ECG screening in RFL athletes, should account for sex, age group, and training influences.

## 1 Introduction

Cardiac electrical adaptations occur in response to chronic exercise training and can be identified through electrocardiogram (ECG) changes (Drezner et al. 2017). Sudden cardiac death (SCD) is the leading cause of mortality in athletes (Harmon et al. 2015) and pre‐participation cardiac screening (PPCS) using a 12‐lead ECG is widely advocated for prevention (Han et al. 2023). However, knowledge gaps exist regarding ECG changes within specific athletic populations, including paediatric and female athletes, as well as in sport‐specific contexts such as Rugby Football League (RFL).

Cardiac remodelling to structured exercise occurs in paediatric athletes, with increased ventricular function, chamber size, and cardiac output being observed (Unnithan et al. 2024, 2018). These adaptations contribute to ECG changes, complicating the distinction between physiological and pathological findings (Fanale et al. 2024). PPCS in paediatric athletes has identified conditions linked to SCD (Fritsch et al. 2017), but with low reported incidence (0.6 per 100,000 per year) (Sarto et al. 2023). Most PPCS research is based on adults, limiting evidence‐based recommendations for paediatric athletes (Riding et al. 2023). Normal paediatric athlete ECGs include unique findings such as T wave inversion in leads V1–V3 (Migliore et al. 2012). Although current ECG interpretation guidelines maximise sensitivity and specificity (Sharma et al. 2018), data specific to informing PPCS in paediatric athletes remain scarce (McClean et al. 2018).

Research on cardiac adaptation in female athletes has received less attention than male counterparts. Males more frequently exhibit sinus bradycardia, isolated QRS voltage criteria for left ventricular hypertrophy (LVH), and longer QRS durations than females (Corici et al. 2018). There is uncertainty as to whether sex differences also exist in RFL athlete ECGs.

Therefore, this study examined ECG findings in RFL athletes, assessing the impact of sex and age group. We hypothesised that male and adult RFL athletes would present with more training‐related ECG adaptations, which could inform future PPCS guidelines.

## 2 Methods

### 2.1 Participants

A total of 161 RFL athletes (65% males and 35% females) aged 14–33 years old participated in the study. Paediatric age group athletes were defined as athletes < 18 years old and adult age group athletes were defined as athletes ≥ 18 years old. Participants were excluded if they presented with an abnormal ECG in accordance with International Criteria (Sharma et al. 2018). Four participants in total were found to have abnormal ECG findings and thus excluded from the analysis. Of these, two participants were excluded due to the presentation of Wolff–Parkinson–White syndrome, a further two participants were excluded with T‐wave inversion in the anterolateral leads.

### 2.2 Experimental Design

The study employed a cross‐sectional design integrated with the RFL athletes' annual off‐season PPCS, which was conducted towards the end of the pre‐season while participants were in full training. Participants were asked to complete a demographic and health questionnaire followed by measurements of height, weight and brachial artery blood pressure. Finally, all participants underwent a 12‐lead ECG. Ethics approval was obtained from the Liverpool John Moores University Ethics Committee (ethics approval number: M23_SPS_3414).

### 2.3 Anthropometric and Blood Pressure Assessment

Body mass measurements were taken using standard calibrated scales (Seca 799, Seca, Birmingham, United Kingdom) and measured to the nearest 1 kg. Height was measured to the nearest 1 cm with a stadiometer (Seca 213, Seca, Birmingham, United Kingdom). Resting brachial artery pressure was measured while the participant was sitting after a 5‐min rest using a manual sphygmomanometer.

### 2.4 12‐Lead ECG

The 12‐lead resting ECG was conducted in a relaxed, supine position. The ECG procedure adhered to established standards (Jevon 2010) and was performed using a commercially available system (Seca CardioPad‐2, Birmingham, UK). ECGs were then analysed by members of the research team and reviewed by a consultant cardiologist. Continuous parameters were extracted from the ECG and included heart rate (HR), P wave duration, PR interval, QRS duration, QTc interval, P axis, QRS axis, T axis and the summation of QRS voltages in V1 and V5 as per criteria for right ventricular hypertrophy (RVH) and LVH (Sokolow and Lyon 1949a, 1949b). The ECGs were also examined in accordance with international criteria for normal training‐related findings, including incomplete right bundle branch block (RBBB), early repolarisation, black athlete repolarisation variant, juvenile T wave pattern, sinus bradycardia, sinus arrhythmia, ectopic atrial rhythm, junctional escape rhythm, first‐degree AV block and Mobitz Type I (Wenckebach) second‐degree AV block. ECGs were also examined for borderline ECG findings including left and right atrial enlargement (LAE/RAE), left and right axis deviation (LAD/RAD) and complete RBBB (Sharma et al. 2018). The recordings were printed onto standard ECG paper and a digital copy was stored securely for further analysis.

### 2.5 Training Questionnaire

Participants completed a training questionnaire to capture the number of years of training and current training hours per week.

### 2.6 Statistical Analysis

Continuous data were presented as mean ± standard deviation (SD), whereas nominal data were expressed as a percentage. Descriptive statistics were used to present participant demographics and incidence of training‐related changes. Two‐way between‐subjects analysis of variance tests were conducted to compare continuous ECG parameters and participant demographics between male and female as well as paediatric and adult age group RFL athletes. Chi‐squared tests were used to compare training‐related ECG findings between groups when nominal or ordinal data were reported.

As a subsequent post‐hoc analysis in adult athletes only, due to much greater variance in age within these groups, we further controlled for the impact of weekly training exposure between males and females. A one‐way analysis of covariance was performed to determine if weekly training hours influenced sex differences in continuous ECG parameters among adult athletes only. Additionally, in nominal or ordinal data outcomes a binary logistic regression was performed to assess the relationship between sex and training‐related ECG findings, with weekly training hours included as a covariate. The statistical significance was set at *p* < 0.05. All data were analysed using SPSS (Version 29, SPSS, Chicago IL).

## 3 Results

### 3.1 Participant Characteristics

Participant characteristics for all RFL athletes are included in Table 1.

**TABLE 1:** Participant demographics.

Baseline measurement	Male paediatrics (*n* = 57)	Female paediatrics (*n* = 18)	Male adults (*n* = 45)	Female adults (*n* = 37)
Age (Years)^c^	16 ± 1	16 ± 1	23 ± 5	23 ± 5
Height (cm)^b^	179 ± 7	166 ± 8	183 ± 5	167 ± 9
Weight (kg)^b^ ^,^ ^c^	81 ± 11	68 ± 11	94 ± 11	77 ± 11
SBP (mmHg)^a^ ^,^ ^c^	121 ± 8	113 ± 7	120 ± 7	122 ± 14
DBP (mmHg)	73 ± 7	72 ± 7	73 ± 7	74 ± 7
MAP (mmHg)^a^ ^,^ ^c^	97 ± 6	93 ± 5	97 ± 6	98 ± 9
Years trained^c^	10 ± 2	10 ± 4	13 ± 3	13 ± 7
Training hours per week^b^ ^,^ ^c^	12 ± 6	7 ± 3	16 ± 5	11 ± 5

*Note:* Data presented as means ± SD.

Abbreviations: DBP = diastolic blood pressure, MAP = mean arterial pressure, SBP = systolic blood pressure.

^a^ Significant interaction.

^b^ Significant main effect for sex.

^c^ Significant main effect for age.

### 3.2 The 12‐Lead ECG

#### 3.2.1 Borderline ECG Findings

Of the borderline ECG findings, LAE was found in 1 Paediatric athlete only. No other borderline ECG findings were found in any RFL athlete.

#### 3.2.2 Impact of Sex

**FIGURE 1:**
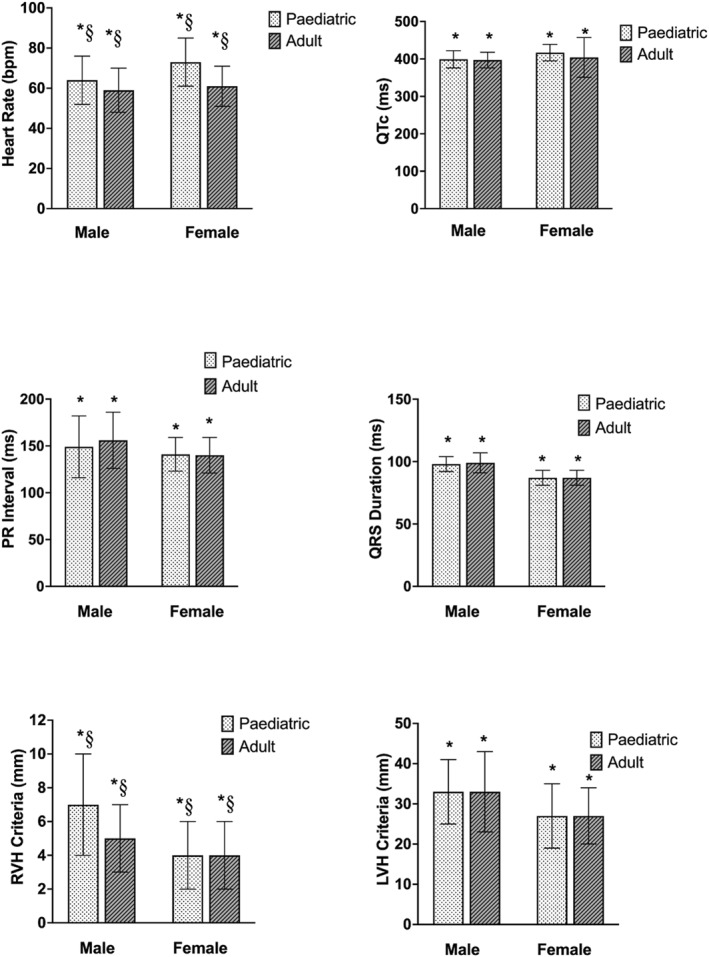
ECG findings in RFL athletes, * = significant main effect for sex, § = significant main effect for age. RVH indicated by RV1 + SV5 or SV6 > 11 mm and LVH indicated by SV1 + RV5 or RV6 > 35 mm.

The ECG parameters and prevalence of training‐related ECG findings are presented in Tables 2 and 3. Significant main effects for sex were observed. Females presented a higher HR (67 ± 11, range: 44–93 vs. 62 ± 11, range: 33–91 bpm; *p* < 0.001) and QTc interval (408 ± 45, range: 352–460 vs. 398 ± 22, range: 352–451 ms; *p* = 0.028) compared to males. Males had a longer PR interval (152 ± 32, range: 60–208 vs. 140 ± 19, range: 110–182 ms; *p* < 0.001), QRS duration (98 ± 7, range: 84–126 vs. 87 ± 6, range: 74–102 ms; *p* < 0.001) and presented increased voltage criteria for RVH (6 ± 3, range: 1–21 vs. 4 ± 2, range: 1–12 mm; *p* < 0.001) and LVH (33 ± 9, range: 16–63 vs. 27 ± 7, 10–45 mm; *p* < 0.001) compared to females. There were no significant main effects for sex on P wave duration, P axis, QRS axis or T axis (Figure 1).

**TABLE 2:** Continuous ECG parameters for RFL athletes.

ECG parameter	Male paediatrics (*n* = 57)	Female paediatrics (*n* = 18)	Male adults (*n* = 45)	Female adults (*n* = 37)
HR (bpm)^b^ ^,^ ^c^	64 ± 12	73 ± 12	59 ± 11	64 ± 10
P wave duration (ms)	96 ± 21	94 ± 10	99 ± 21	91 ± 19
PR interval (ms)^b^	149 ± 33	141 ± 18	156 ± 30	140 ± 19
QRS duration (ms)^b^	98 ± 6	87 ± 6	99 ± 8	87 ± 6
QTc interval (ms)^b^	399 ± 23	417 ± 22	397 ± 21	404 ± 53
P axis (°)	38 ± 5	44 ± 16	36 ± 27	35 ± 28
QRS axis (°)	65 ± 33	66 ± 23	68 ± 28	65 ± 19
T axis (°)	38 ± 15	35 ± 12	30 ± 13	33 ± 22
Voltage criteria for RVH (mm)^a^ ^,^ ^b^ ^,^ ^c^	7 ± 3	4 ± 2	5 ± 2	4 ± 2
Voltage criteria for LVH (mm)^b^	33 ± 8	27 ± 8	33 ± 10	27 ± 7

*Note:* Data presented as means ± SD. RVH indicated by RV1 + SV5 or SV6 > 11 mm and LVH indicated by SV1 + RV5 or RV6 > 35 mm.

^a^ Significant interaction.

^b^ Significant main effect for sex.

^c^ Significant main effect for age.

**TABLE 3:** Prevalence of normal training‐related ECG findings in RFL athletes.

ECG finding	Male paediatrics (*n* = 57)	Female paediatrics (*n* = 18)	Male adults (*n* = 45)	Female adults (*n* = 37)
Increased QRS voltage^a^	24 (15.28)	3 (1.91)	14 (8.92)	5 (3.18)
Incomplete RBBB	8 (5.09)	0 (0)	3 (1.91)	3 (1.91)
Early repolarisation^a^	15 (9.55)	0 (0)	19 (12.10)	4 (2.55)
Black athlete repolarisation variant	1 (0.64)	0 (0)	0 (0)	0 (0)
Juvenile T‐Wave pattern	0 (0)	0 (0)	0 (0)	0 (0)
Sinus bradycardia	23 (14.65)	0 (0)	22 (14.01)	14 (8.92)
Sinus arrhythmia	10 (6.37)	0 (0)	10 (6.37)	6 (3.82)
Ectopic atrial rhythm	2 (1.27)	0 (0)	0 (0)	1 (0.64)
Junctional escape rhythm	0 (0)	0 (0)	1 (0.64)	1 (0.64)
First degree AV block	1 (0.64)	0 (0)	0 (0)	0 (0)
Mobitz Type I (Wenckebach) Second degree AV block	0 (0)	0 (0)	0 (0)	0 (0)

*Note:* Data presented as *n* (%) of RFL athletes.

^a^ Significant association between sex and training‐relating ECG finding observed.

There was a greater prevalence of early repolarisation (33 vs. 7%; *p* < 0.001) in males compared to females. There were no significant associations between sex and presence/absence of incomplete RBBB, sinus bradycardia, sinus arrhythmia, ectopic atrial rhythm, black athlete repolarisation variant, juvenile T‐wave pattern, junctional escape rhythm, first‐degree atrioventricular block and Mobitz Type I (Wenckebach) second‐degree AV block (Table 3).

#### 3.2.3 Impact of Age Group

Significant main effects for age group were observed. Adult athletes had trained for more years (13 ± 5, range: 2–27 vs. 10 ± 2, range: 3–14 years; *p* < 0.001) than Paediatric athletes. Paediatric athletes presented a higher HR (66 ± 13, range: 33–93 vs. 61 ± 10, range: 42–87 bpm; *p* < 0.001) and increased voltage criteria for RVH (7 ± 3, range: 1–12 vs. 5 ± 2, range: 1–21 mm; *p* = 0.015) when compared to adult athletes. However, there were no significant main effects observed for age group on P wave duration, PR interval, QRS duration, QTc interval, P axis, QRS axis, T axis and criteria for LVH (Figure 1).

There were no significant associations observed between age group and presence/absence of incomplete RBBB, early repolarisation, sinus bradycardia, sinus arrhythmia, ectopic atrial rhythm, black athlete repolarisation variant, juvenile T‐wave pattern, junctional escape rhythm, first AV block and Mobitz Type I (Wenckebach) second‐degree AV block (Table 3).

#### 3.2.4 Interaction Between Sex and Age Group

Only voltage criteria for RVH was significant for interaction of sex and age group (F_1, 153_ = 5.61, *p* = 0.019). Male paediatric athletes presented increased RVH voltage compared to male adult athletes (7 ± 3, range: 1–21 vs. 5 ± 3, range: 1–10 mm), while female paediatric and adult athletes had similar values (both 4 ± 2, range: 1–12 mm).

### 3.3 Adult Athletes and Training Exposure

#### 3.3.1 Impact of Weekly Training Hours

Within the adult athletes, males reported a higher number of weekly training hours (16 ± 5, range: 8–30 vs. 11 ± 5, range: 4–22 h; *p* < 0.001) than females with no other differences in training exposure. There was a significant main effect for sex in adult athletes when controlling for weekly training hours. Males presented a longer PR interval (156 ± 32, range: 114–194 vs. 141 ± 19, range: 112–182 ms; *p* = 0.037) and QRS duration (99 ± 7, range: 84–126 vs. 87 ± 7, range: 76–102 ms; *p* < 0.001). However, after controlling for weekly training hours there was no significant sex difference on any other continuous ECG parameter. In addition, logistic regression analysis indicated that sex, when controlled for weekly training hours, had no impact upon the nominal or ordinal training‐related ECG findings (all *p* > 0.05).

## 4 Discussion

The main findings from this study were: (1) across paediatric and adult RFL athletes, males exhibit longer PR intervals, QRS durations, and more voltage criteria for RVH and LVH compared to female athletes, while females demonstrate increased HR and QTc intervals; additionally, adult athletes exhibit lower resting HR and reduced criteria for RVH compared to paediatric athletes, (2) males show increased QRS voltage and early repolarisation more frequently than females across both paediatric and adult age groups and (3) when controlling for weekly training hours in adult athletes, males present longer PR intervals and QRS durations, while differences in other continuous, ordinal or nominal training‐related ECG findings are absent.

### 4.1 Impact of Sex

The impact of sex on ECG findings linked to athletic adaptation in RFL athletes aligns with research carried out on other athlete populations (Bessem et al. 2017; Corici et al. 2018; Storstein et al. 1991). A study involving 315 athletes representing a variety of sporting disciplines found that males presented with an increased QRS duration and met the criteria for RVH more frequently than female athletes, while females exhibited a higher QTc interval and increased resting HR compared to males (Corici et al. 2018). Additionally, males had a greater incidence of sinus bradycardia and females showed a higher incidence of sinus arrhythmia (Corici et al. 2018). The current study also found male RFL athletes present a significantly greater PR interval, and a higher incidence of early repolarisation compared to females. Furthermore, while Corici et al. (2018) found females to have a greater incidence of sinus arrhythmia, no significant associations between sex and sinus arrhythmia were observed in the current study. This suggests that sex differences in normal training‐related ECG findings may be different dependant on sporting discipline.

There were observed sex differences in ECG findings shown not to be impacted by variations in weekly training hours. This indicates that the increased PR and QRS durations observed in males are directly associated with sex. Oestrogen and progesterone are likely key factors in observed ECG differences, as they are known to contribute to increased HR and QTc intervals along with shorter PR intervals and QRS durations in females (Gowd and Thompson 2012). This aligns with the findings of this study where we observe similar patterns in females athletes. However, despite fluctuations in oestrogen and progesterone levels across different phases of the menstrual cycle, ECG parameters remain stable in physically active females (Morrison et al. 2023) suggesting that hormonal changes may not significantly alter the ECG during athletic activity. Additionally, a negative linear correlation between HR and PR interval has been established (Carruthers et al. 1987). Therefore, the lower HR observed in male athletes suggests an increased vagal tone and likely reflects the increased PR intervals in this group of RFL athletes (Aro et al. 2014; Capilupi et al. 2020).

Interestingly, the absence of sex differences in ECG findings across all other parameters after controlling for weekly training hours, is a novel observation. Notably, previous studies examining sex differences in ECG findings have not accounted for training hours in their analysis, making this a unique contribution to the existing literature (Bessem et al. 2017; Corici et al. 2018). This contrasts with findings in non‐athlete populations, where females display increased HR and QT intervals compared to males (Albert et al. 1996; Mason et al. 2007). However, the increased QRS durations and PR intervals found in male RFL athletes have also been found in non‐athletes (Mason et al. 2007; Rautaharju et al. 2014) suggesting these differences may not be exclusive to athletes. Therefore, the findings from the current study indicate that training hours have a greater impact on physiological ECG adaptations than sex on RFL athletes. ECG parameters such as HR, P wave duration and QTc interval are known to decrease with increased training duration (Dawkins et al. 2020). Therefore, future research should consider sex differences in training exposure to determine whether ECG adaptations are more closely associated with sex or training volume.

### 4.2 Impact of Age

Although the differences were not as profound across age groups as sexes. An Increased HR and RVH criteria for paediatric athletes was observed. Differences in HR are to be expected due to the adult athletes increased training duration. Additionally, a meta‐analysis found the prevalence of RVH in paediatric athletes being 9.8% (McClean et al. 2018). Paediatric athletes are known to exhibit RV dominance, as they are yet to fully develop adult ventricular mass (Molinari et al. 2017). This can alter the direction and propagation of the electrical impulse resulting in RVH criteria being met on the ECG despite the absence of a cardiomyopathy (Zaidi et al. 2013).

### 4.3 Future Directions

The observed ECG findings in RFL athletes may have important implications for future PPCS. Although the normal male RFL athlete ECG has previously been described (Forsythe et al. 2016), this study enhances current knowledge by incorporating data from females and paediatric athletes. The study also fills a gap in the lack of sport‐specific research on ECG changes in female athletes (Panhuyzen‐Goedkoop et al. 2024). These data from female and paediatric athletes can provide insight for the development of more comprehensive and tailored PPCS guidelines (Sharma et al. 2018). Moreover, the absence of significant sex differences in ECG findings after accounting for weekly training hours highlights the impact of training adaptation on ECG parameters. However, international ECG interpretation recommendations do not account for variations in training volume, which may compound any differential diagnosis (Sharma et al. 2018). Despite controlling for training hours, significant sex differences in QRS duration and PR interval persisted. Since current guidelines do not differentiate between sex when defining normative ranges for these parameters (Sharma et al. 2018), our findings suggest that further investigation into sex‐specific ranges may be necessary for future recommendations. Additionally, this research supports lowering the classification threshold for profound first degree AV block to PR interval of 300 ms in paediatric athletes (Ragazzoni et al. 2023), as no athlete in our study exceeded this value.

### 4.4 Limitations

Although the current study provides valuable data which can be used to inform future PPCS for RFL athletes, there were some limitations. The majority of the participants were white athletes with 5 pacific islanders, 11 individuals with a mixed ethnic background (white and black African/Caribbean). Previous research has shown no differences in normal ECG criteria between white and Pacific Islander RFL athletes (Johnson et al. 2018). However, evidence suggests that athletes with a mixed ethnic background exhibit specific ECG variation similar to black athletes, including differences in repolarisation (Malhotra et al. 2021; Papadakis et al. 2011). Moreover, all participants were recruited from the same RFL club which could limit the external validity to other RFL athletes who follow different training regimes. Furthermore, comparisons based on age may reflect differences in training duration rather than true age‐related effects, as older athletes often have more extensive training histories. Additionally, sex differences may be influenced by professional status, as male RFL athletes were professional while female RFL athletes were semi‐professional. Maturity status data were not collected in this study. Given the wide age range of the adolescent athletes, future studies should consider accounting for maturity status (McClean et al. 2018). Finally, the absence of echocardiographic data limits further insight (McClean et al. 2018).

## 5 Conclusion

This study highlights significant differences in ECG findings among RFL athletes based on sex and age. Male athletes showed longer PR intervals and increased QRS voltage/duration, while females exhibited higher resting HR and QTc intervals. Adult athletes displayed a reduced HR and RVH criteria compared to paediatric athletes. However, when controlling for variations in weekly training exposure between sexes, only differences between PR and QRS intervals were observed. These results highlight the need for consideration of an RFL athletes' sex and age/training duration. Future research should consider a broader range of athletes to further explore sport‐specific training‐related ECG changes.

## Ethics Statement

The study was approved by the Liverpool John Moores University Ethics Committee (approval number: M23_SPS_3414). All procedures performed in the study were in accordance with the ethical standards of the institutional research committee and with the 1964 Helsinki Declaration and its later amendments or comparable ethical standards.

## Consent

Informed consent was obtained from all participants involved in the study, including parental consent for participants under the age of 18.

## Conflicts of Interest

The authors declare no conflicts of interest.

## Permission to Reproduce Material From Other Sources

No previously published material was reproduced in this study.

## Data Availability

The data that support the findings of this study are available on request from the corresponding author (Prof. David Oxborough). The data are not publicly available due to privacy or ethical restrictions.
